# Expertise among professional magicians: an interview study

**DOI:** 10.3389/fpsyg.2014.01484

**Published:** 2014-12-23

**Authors:** Olli Rissanen, Petteri Pitkänen, Antti Juvonen, Gustav Kuhn, Kai Hakkarainen

**Affiliations:** ^1^School of Applied Educational Science and Teacher Education, University of Eastern FinlandSavonlinna, Finland; ^2^Department of Psychology, Goldsmiths, University of LondonLondon, UK; ^3^Institute of Behavioural Sciences, University of HelsinkiHelsinki, Finland

**Keywords:** expertise, expertise in magic, performing, professional satisfaction, reflection, creativity, professional magician

## Abstract

The purpose of the present investigation was to analyse interviews of highly regarded Finnish magicians. Social network analysis (*N* = 120) was used to identify Finland's most highly regarded magicians (*N* = 16). The selected participants' careers in professional magic and various aspects of their professional conduct were examined by relying on semi-structured interviews. The results revealed that cultivation of professional level competence in magic usually requires an extensive period of time compared with other domains of expertise. Magic is a unique performing art and it differs from other professions focusing on deceiving the audience. A distinctive feature of magical expertise is that the process takes place entirely through informal training supported by communities of magical practitioners. Three interrelated aspects of magical activity were distinguished: magic tricks, performance, and audience. Although magic tricks constitute a central aspect of magic activity, the participants did not talk about their tricks extensively; this is in accordance with the secretive nature of magic culture. The interviews revealed that a core aspect of the magicians' activity is performance in front of an audience that repeatedly validates competence cultivated through years of practice. The interviewees reported investing a great deal of effort in planning, orchestrating, and reflecting on their performances. Close interaction with the audience plays an important role in most interviewees' activity. Many participants put a great deal of effort in developing novel magic tricks. It is common to borrow magic effects from fellow magicians and develop novel methods of implementation. Because magic tricks or programs are not copyrighted, many interviewees considered “stealing” an unacceptable and unethical aspect of magical activity. The interviewees highlighted the importance of personality and charisma in the successful pursuit of magic activity.

## Introduction

Magicians have acquired a unique set of skills that allow them to create illusions of the impossible, and in recent years scientists have become interested in exploring this expertise to further our understanding of cognition (Kuhn et al., 2008; Rensink and Kuhn, 2014). To date, relatively little is known about how this expertise develops. Magic differs significantly from other domains of expertise (e.g., music, stand-up comedy) in that most learning takes place in personal practice that is embedded within informal social networks (Rissanen et al., 2010, 2013), and thus with very little formal training (i.e., magic schools). Without formal training, it is difficult to determine the skills needed to perform magic well.

In most other domains (e.g., sport, chess), expertise can be objectively measured through formal competitions. While there are several national and international magical competitions, it is commonly known that most of the best magicians do not participate in these competitions. Moreover, the skills and techniques required to win a magic competition often vary from those used by professional magicians. For example, although fellow magicians can be deceived, it is much harder to deceive people who have sophisticated knowledge about conjuring methods (Lamont and Wiseman, 1999). Moreover, the tricks that are typically used to fool fellow magicians are often very different from the ones performed to entertain lay people. When performing for fellow conjurers, magicians typically use methods that are far more technical and impressive (e.g., difficult sleight of hand, difficult mental skills, complex methods), than when performing for a lay audience. A further problem in studying magical expertise is that conjuring involves a wide variety of skills. For example, a magician must have a wide range of psychological skills, such as the ability to use external cues and signs (e.g., reactions, applause, verbal feedback) to infer about the audience's mental state (e.g., experience of the effect, whether they detected the method). Similarly, the magicians must be able to use psychological techniques to effectively misdirect the audience, and thus prevent the audience from noticing the method used to create the effect. Many of these misdirection techniques have been documented and described (e.g., Kuhn et al., 2014), and effective deception requires a solid understanding of these psychological principles. Other skills involve motor skills (e.g., sleight of hand), technical insights (e.g., abstract knowledge of magic techniques), as well as performance specific techniques (e.g., comedy, dance).

We consider the pursuit of magic as a specific form of expertise that involves sophisticated skills and well-organized professional knowledge of conjuring performed at the highest national and international standard (Ericsson and Charness, 1994; Chi, 2006; Ericsson et al., 2009). Expertise has been investigated in many fields such as science, arts, and sports (Ericsson, 1996, 2003, 2006; Ericsson and Starkes, 1996; Faulkner et al., 1998). Magicians are entrepreneurs who need to master diverse bodies of skills and competencies.

Although magic has some commonalities with other performing arts, it relies heavily on secretive knowledge and competence, which is disseminated within a network of experienced magicians. Newcomers become magicians by participating in their “community of practice” (Lave and Wenger, 1991) sharing knowledge and fostering conjuring skills, and the expertise develops through the guidance of experts. Advanced magical knowledge can only be accessed once junior magicians have established trust-laden relations with practicing magicians. Developments in social media and the Internet have substantially changed the knowledge transfer amongst magicians. The sharing of online videos of performances and magic tutorials has had profound impacts on how new tricks and techniques are learnt. For example, it is far easier to learn complex sleight of hand and misdirection techniques by observing a magician on video, than by reading abstract descriptions in a book. Moreover, much of magic relies on subtleties that are difficult to describe in text and thus video resources provide much additional information about techniques as well as presentation styles that were previously unavailable. Magic chat rooms and online videos allow magicians to exchange ideas and develop new tricks. The Internet has made much of the material more accessible, and it has also led to a rapid acceleration by which new tricks and methods are shared amongst magicians and the general public. Not all of these developments have, however, been positive. These online resources have facilitated the copying of entire magic routine and the easy access of magic material has also facilitated exposure of magic methods to the general public. As such professional magicians can no longer rely on their secret method and must adapt their methods and performance to stand out as a professional performer (Swiss, 2001). Maintaining a high degree of expertise requires the experts to update their knowledge and develop new tricks and entertainment programs.

Performing magic in front of a live audience is the magicians' core activity. According to Ortiz (2006), magical activity involves three elements. The first is the technology of magical methods. It requires magical instruments, for instance, in the form of sleights, gaffs, and psychological ploys that assist in creating a magic effect. Magical instruments and methods enable magicians to prevent the audience from discovering the ways of completing the trick; the resulting secrecy plays an important role in bringing about a magical experience for the audience. Second, it is also essential to have showmanship to highlight the dramatic, emotional, and magical power of the performance. A crucial element between method and showmanship is effect design; that is the astonishing and mysterious leap from the initial to the final condition that is at the core of the magical process. The field of magic is very wide and involves various genres from stage illusions, manipulations, close-up magic, street magic, comedy magic, mentalism, psychological illusionism, theatrical mentalism, and bizarre magic (Landman, 2013). The magic genres are diverging specific effects played for the audience and the performers cultivate corresponding images and brands in relation to the public. Continuous audience feedback from more or less successful performances and personal and collaborative post-performance reflection are important forces that drive development. Achieving a top level skill requires one to enter difficult situations and systematically practice at the upper echelons of one's proximal development rather than only acting in one's zone of comfort (Hatano and Inagaki, 1992; Bereiter and Scardamalia, 1993; Hakkarainen et al., 2004).

The purpose of the current paper was to examine the nature of a professional magician's expertise through a semi-structured interview. We focused on the following four questions:

1. Through what stages does the expertise of a professional magician develop?
2. What are the distinctive features of magical expertise?
3. What is the role of magical tricks, performance, and audience in professional pursuit of magic?
4. To what extent do professional magicians share their achievement and pursue novelty and innovation?

## Methods

### Participants and the context

**Figure 1:**
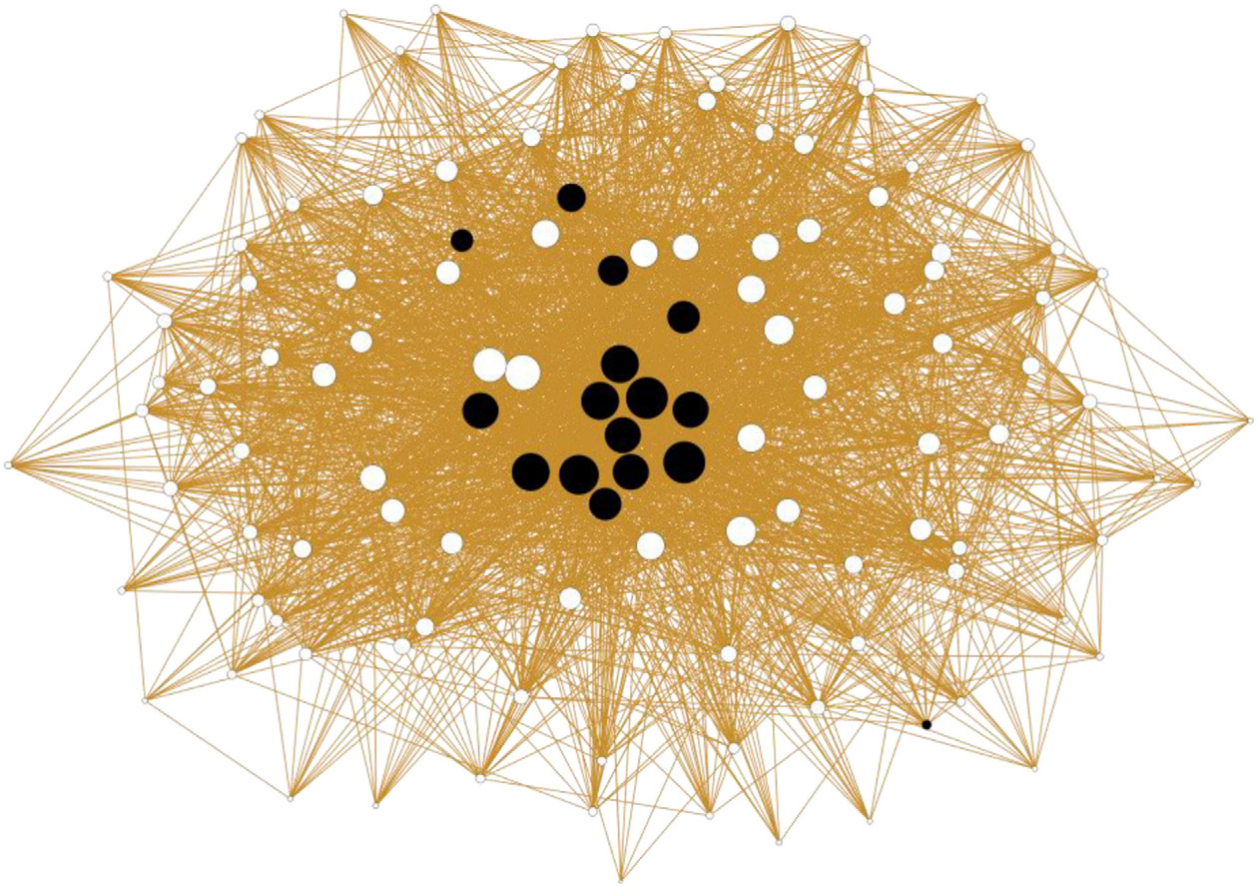
**Social-network graph regarding social recognition of magical expertise (*N* = 120)**.

Data about the magicians' networking relations were collected via questionnaire based on the members of the national magician network. Participants were asked to indicate, in relation to each other, those community members who they rate highly as a performing magician. The questionnaire was submitted to 148 known Finnish magicians who had been identified by the first author and three professional magicians (response rate = 81%). A social network analysis that focused on analysing centrality of the participation was conducted (Borgatti et al., 2002). The magicians' peer evaluations were used to create indicators by nominating respected magicians. Analyses indicated that social recognition was not correlated with age. Figure 1 presents a social-network graph regarding social recognition of magic expertise.

Black nodes represent the interviewed professional magicians (*N* = 16). White nodes represent the other actors of the magical field (*N* = 104). The size of nodes is determined according to in-degree regarding professional recognition.

On the basis of the social-network analysis (*N* = 120), 16 key experts were selected for a semi-structured theme interview using several criteria. We contacted 17 of the most highly rated magicians, though three were unavailable for an interview. Most of the magicians are males and there are only a few female ones. Because of that, we decide to include to the interview sample also two female magicians. Although one of them was peripherally located, she was selected for interview because of being considered as a rising star excelling in national and international competitions. All participants were professionally active, healthy, and successful in national and/or international competitions. In order to protect the anonymity of the participants (M1–M16), some of the information (e.g., gender) is not reported in the present article. The interviews were carried out in Finnish and the data reviewed by all Finnish authors. We do not reveal identities of the participants because interviewees were promised that the interview data will be reported anonymously.

### Interview method and analysis of data

Various aspects of the selected magicians' professional expertise were examined through a semi-structured interview (Kvale and Brinkmann, 2009). In accordance with an egocentric network interview (Marsden, 2002; Palonen, 2006; Hogan et al., 2007), the participants were asked to draw a timeline of their professional careers. In addition, they were asked to name important people for their career; this was used to ground interview questions regarding collaborators and other significant networking partners. The interviews were usually carried out at the participants' homes and took place between April 2009 and May 2011. The interviews took from 57 min to 3 h and 37 min, depending on the length of the individual's career and the articulacy of the interviewee.

The interviews were transcribed word by word and analyzed qualitatively using ATLAS.ti 6.2 (see atlasti.com). This program allows the researcher to present the transcribed interview text in one column and thus identify and mark qualitatively differing text segments. The code of the text segment is presented in another column. Working with these two columns representing, respectively raw interview data and associated coding, it is possible to refine the coding system across successive cycles of analysis. Initially, the interviews were read several times to get an overview of central contents and themes. Next, text segments relevant to purposes of the present investigation were categorized into the same hermeneutic category to exclude irrelevant material, such as detailed personal recollections of one's career. In order to identify the central themes, we created ATLAS.ti codes for text segments corresponding to the main interview and research questions. If an interviewee did not answer an interview question in the associated context, it was searched from other parts of the transcribed interview and coded accordingly. If a text segment did not correspond to the interview questions, it was given a code describing the content as comprehensively as possible. Across the analysis new emergent code, such as internet, audience and performance was generated. The main themes identified consisted of: (1) orientation to magic, (2) professional development and personal networks, (3) professional profile and the development of expertise, (4) performance and relation to audience, (5) creation of novelty and innovation. Each of the categories was analyzed in detail to identify sub-themes. The data were categorized independently by two coders who repeatedly met, compared their observations, and sorted out disagreements. From the coded data, we identified reoccurring themes and examined frequencies of corresponding text segments. Subsequently, the data were analyzed to find common themes and distinguishing features in accordance with a theory-informed, data-driven approach (Frank, 1995, 1996, 1998; Fereday and Muir-Cochrane, 2006). Interesting observations, occurring during the analysis, were documented in associated ATLAS.ti memos. Finally, the data were screened for quotations and compressed descriptions regarding various aspects of magician activity. The quotations were selected in researcher meetings to describe the findings by using respondents' own words. In the interviews, the participants reported their first contact with magic, the development of a professional profile, growth of their professional knowledge and competence, and reflected both on importance of old traditions and development of new magic tricks and programs. The analysis focused on examining strategies and experience performance, experienced professional satisfaction, the development of interviewees' professional profiles, and their creation of new tricks and performances. The egocentric networks were visualized by Cytoscope program (2012) that integrated the presentation of all interviewees' partially overlapping personal networks and structures of their relations. Table 1 (Appendix) presents a summary of the interview data analyzed.

## Results

The results section is organized as follows: First, we will examine development of magical stage expertise, focusing on the magician's career. Second, we will analyse networking partners and factors related to pursuit of magic at the professional level. Third, we will address central aspects of magical expertise according to the interviewees' accounts. Finally, we will reflect on the interviewees' overall idea of being a professional magician and its essential dimensions on the basis of the analyzed data.

### Trajectories for becoming a professional magician

**Figure 2:**
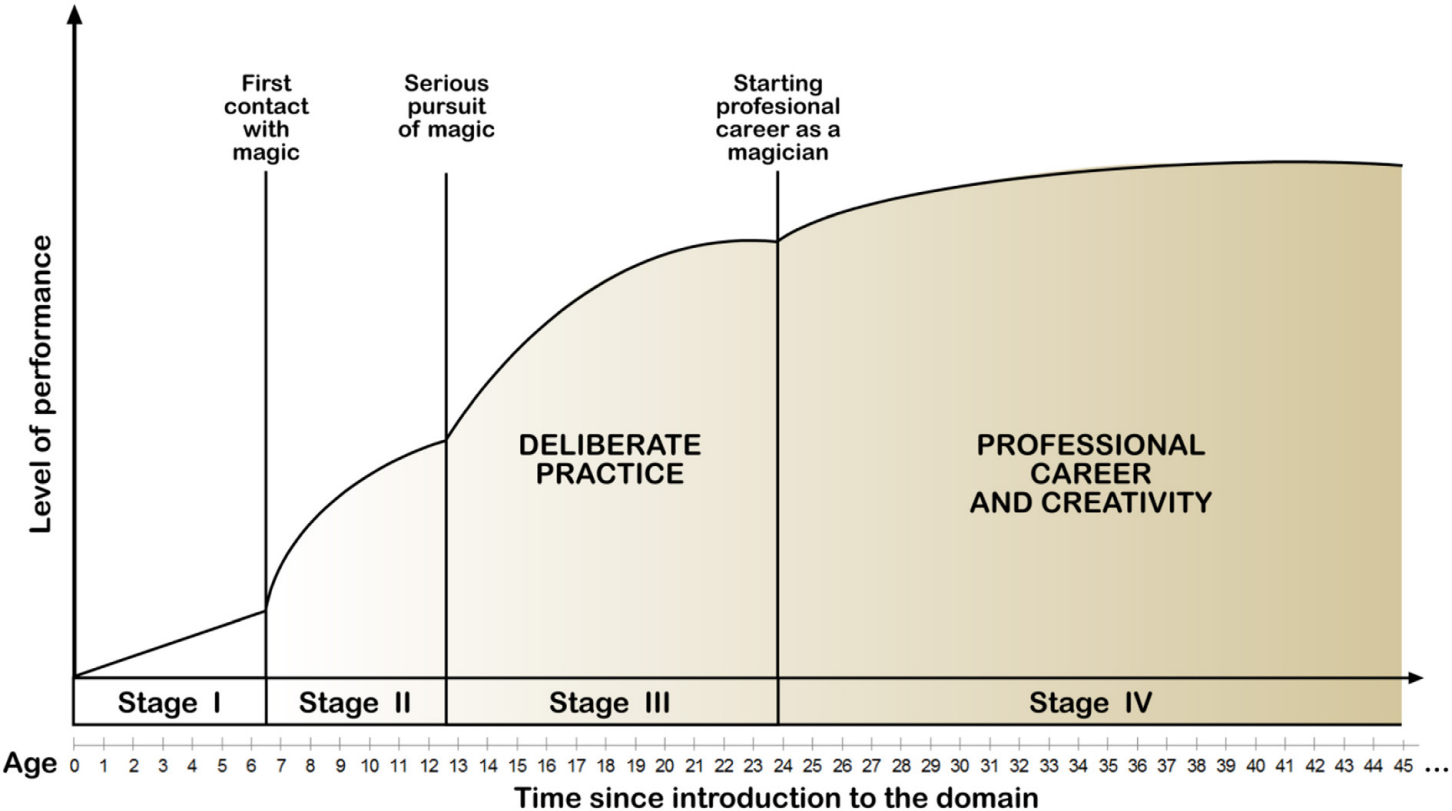
**Trajectories for becoming a professional magician (adapted from Ericsson, 2003) as retrospectively reconstructed on the basis of the present interview data**. Characterizes average developmental trajectory of the interviewees (*N* = 16) based on their retrospective accounts. Stage I: From birth to first contact with magic (*M* = 7; *SD* = 2.5); Stage II: Serious interest (*M* = 6; *SD* = 4.9). Stage III: Deliberate practice (*M* = 11; *SD* = 5.5). Stage IV: Reaching a professional level and pursuing further professional development (*M* = 23; *SD* = 9.8).

The interviewees (*N* = 16) were asked to reflect on their trajectories for becoming professional magicians. Figure 2 illustrates different stages of the developing expertise in magic from first contact (I), time of starting a serious pursuit of magical expertise (II), beginning of a professional career (III), and establishing a stable professional career (IV).

The interviewees reported having their first contact with magic, on average, at age seven; all except one were between 4 and 9 years old. The first experience involved watching a magic show, experiencing a magic trick or reading a magic book; interest in magic emerged from such an influential experience encouraging the first efforts in enjoying performing magic tricks and gradually developing competencies (Bloom, 1985; Ericsson, 1996). In stage II, the interviewees' serious interest arose between the ages of 7–13 leading to a more deliberate pursuit of skill development. Initially, the development of competency was fast and involved seeking support from more competent peers and adult experts, such as fellow magicians, professionals, and personal mentors. Intensive, deliberate practice was initiated, on average, at the age of 13. In accordance with the 10-year rule (Ericsson et al., 1993), participants reported having deliberately practiced magic for more than 10 years (*M* = 11.1, *SD* = 5.5). When reaching a relatively high level of expertise (stage III), participants were able to initiate professional careers as magicians. On average, professional careers started at the age of 24. The youngest professional magician was aged 16, and the oldest was 34.

A great deal of effort was needed to establish a stable career and cultivate an original and distinctive profile as a magician. All respondents working as professional magicians, except for one retiree, have been doing so for 22 years. The development of expertise continuously improves during the career, requiring the continuation of acquiring skills. Participants reported utilizing workshops, occasional courses, lectures, magical clubs, peers, mentors, books, videos, and the Internet when cultivating their craft (Jones, 2011). The Finnish magic associations play an important role by organizing annual workshops, national and international competitions, and publishing a national magic magazine (Jokeri). As indicated below, several respondents emphasized the importance of sustained professional development without which expert level cannot be maintained in a changing environment.

**Figure 3:**
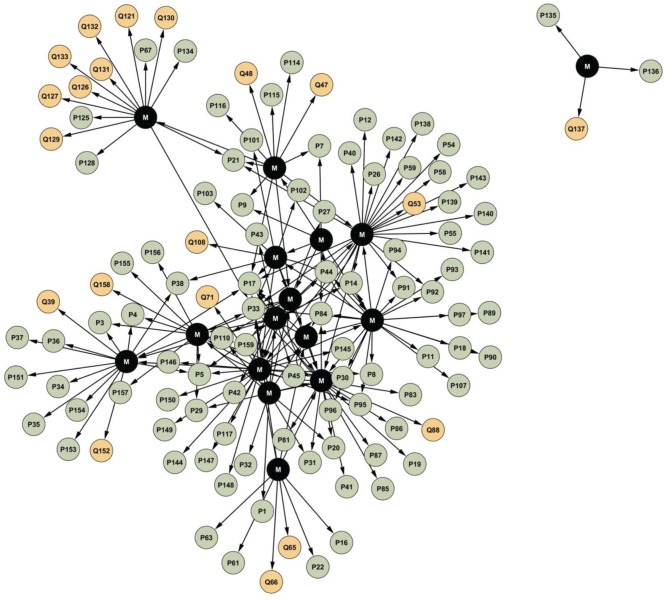
**Egocentric network of the interviewees and people significant for their careers**. M (black nodes) represents the interviewees; P (green nodes) represents magicians significant to a career in magic; Q (yellow nodes) represents other significant persons.

Figure 3 describes egocentric networks of the interviewees and people who have played significant roles in their career. The data revealed that several of the respondents had collaborated with each other during their careers. The interviewees referred to 127 people altogether who had influenced their careers. The networking partners consisted of foreign contacts, persons significant for the development of their careers, masters and mentors who trained them, as well as close colleagues and collaborators. Overall, the Finnish magic community is rather tightly organized around a core consisting of a few central persons, although centralization of the network is not very high. The level of connectivity may be affected by place of residence, age, and professional contacts. Three out of four respondents reported that they had designated mentors or masters who played an important role across their career, especially in the beginning. Some participants established international careers and became famous in other countries after winning international competitions; one of them had a personal network separated from the others. A female magician is located outside of the main body of the network because of having worked in a foreign country (the rising star); this is the reason for having her own network separated from those of the other 15 interviewed magicians. The present investigation reveals that although magicians tend to practice and function individually, they have much contact with fellow magicians and external experts. Beyond magicians, collaborators included an actor, conductor, customer manager, manager, producer, agent, speaker, and theater director. Magicians collaborate by following each other's performances, assessing new tricks, giving feedback on magic shows, and sharing their knowledge and competence. Mutual trust is important for professional development and cultivation of expertise. Currently, mobile connections, social media, and the Internet facilitate professional interaction and sharing of knowledge.

### Professional magicians' central domains of activity

It was noted that a magician's professional expertise develops through deliberate practice (Ericsson et al., 1993, 2007; Ericsson, 1996, 2009). Their multi-faceted competencies require integration of knowledge and skill to support flexible functioning in varying performance situations and environments. The interviewees reported that successful functioning as a magician requires professional passion, building of networking relations, guidance from mentors, tapping into cultural resources of the field, sharing professional know-how, and creating new tricks and programs. Toward that end, professional magicians reported it necessary to cultivate a versatile set of skills and competencies, such as manual dexterity, motoric skills, the capability to read an audience, manipulation skills, working with animals, creativity, personal charisma, and skills of self-reflection.

The interviewees argued that a magician has to master all of the main elements of magic activity; if one of them is defective or does not work, successful professional performance may not be possible. They stated that a magician must have multiple skills and competencies because the profession includes diverse elements, such as the stage presence, marketing, product development and design, sound and lighting design, script writing, props, costumes, and equipment. Magicians need to be flexible and have the ability to cope with expectations of increasingly heterogeneous and demanding audiences. As experts, magicians need well-rehearsed routines, but those are often not enough; they also need to systematically invest in learning new skills and competencies.

**Figure 4:**
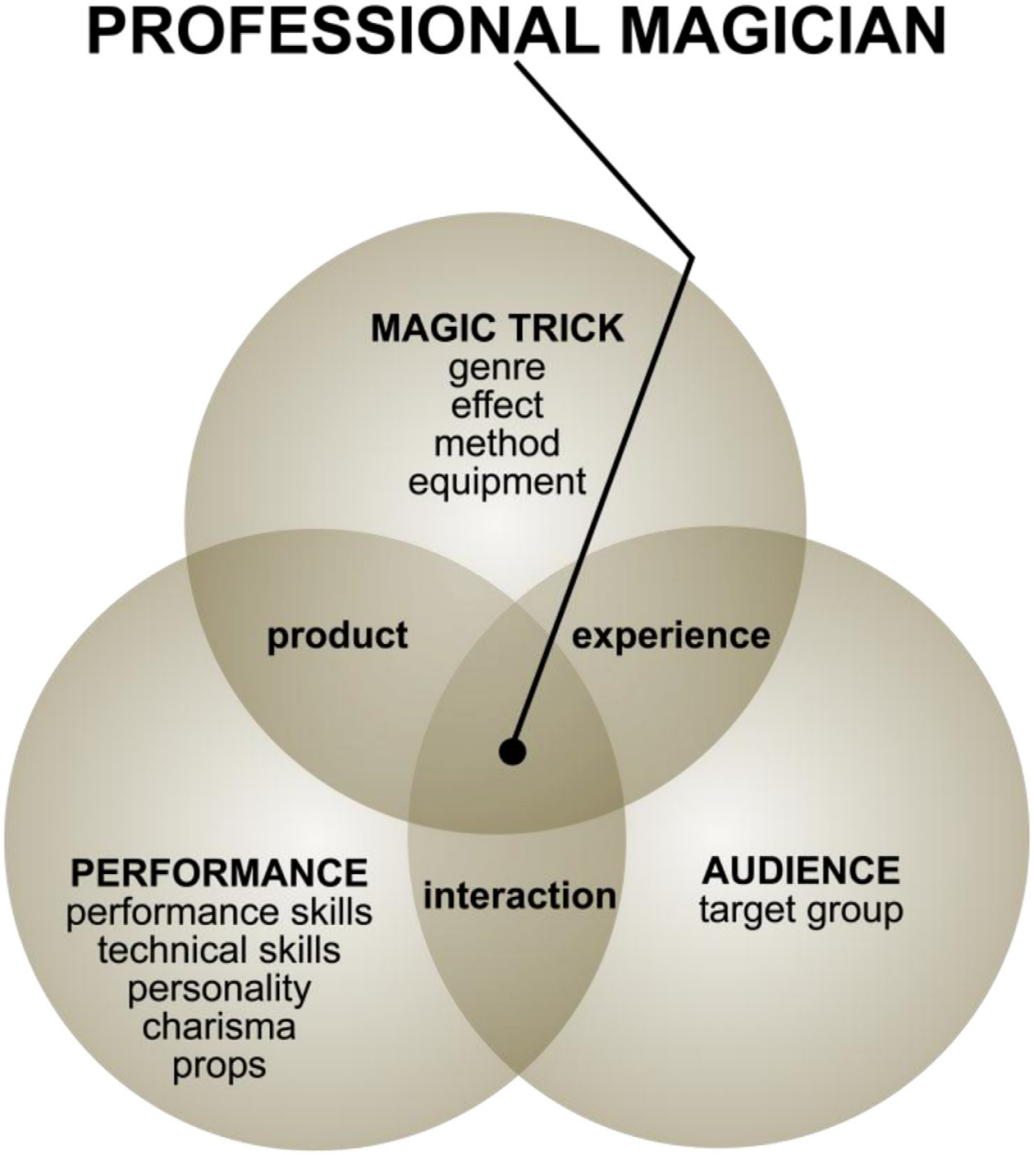
**Central themes regarding magical activity occurring in interviews of highly regarded magicians**.

On the basis of the qualitative analysis, we categorized the magicians' professional activity according to three core areas: magic tricks, performance, and audience (Figure 4). Designing magic tricks represents the core competency of a magician; magic activity cannot be understood without addressing it. Magic is a performing art; magicians pursue their professional activity by performing magical shows (i.e., product) consisting of a series of tricks and associated performative activities (e.g., stories) in front of an audience. Further, a skilled magician tailors his or her performance according to the audience and functions in close interaction with it. In a successful magical show, the audience, in turn, goes through thrilling experiences. In order to deliver a successful performance, the magician has to take account of and manage a number of different aspects.

#### The magic trick

We asked the interviewees to reflect on various aspects of their activity, including magic tricks. The participants did not, however, talk that much about magic tricks during the interview extensively; this is in accordance with the secretive nature of the magic culture. In addition, magic tricks are basic to the domain and form a self-evident requirement for professional magicians. The interview indicated, further, that individual tricks were not the professional magician's focus. Although a particular key trick may have a significant role in the performance, the interviewees emphasized the importance of the overall magic show. Yet, there is no magic without magic tricks. The magic trick is the basic tool of astonishing the audience. Both mental and manual skills are combined successfully in performing magic.

Magic emerges from an impossible or unexplainable phenomenon which creates a conflict between what the audience thinks is possible and the event they have just been observed (Parris et al., 2009). The spectator tries to solve the puzzle but a skillfully constructed magic routine does not allow the audience to rationally explain what they have observed and experienced. He or she cannot solve the riddle. The magician relies on misdirection, forcing, or illusion techniques depending on the methods of the trick and the desired effects.

According to M11, it is very challenging to come up with a magical effect: “Coming up with an effect is one of the toughest things to do. Almost always if you've got an effect you come up with a method – you may not be satisfied with it but you come up with something. And it's, if we talk about coming up with something new, it's one of the toughest things to do.” M11 tells about ideas that Spanish magician Juan Tamariz has been developing across decades: “Tamariz completed two tricks last summer that he had started working on more than thirty years ago. This goes to show how long it can take to construct these tricks. The process of creating can be such a prolonged birthing process and it can come with a lot of pain, too. So maybe it can be compared to giving birth – it's tough but once it's born, it's a beautiful thing.”

Magicians practice their tricks technically so often that performing them in their programs consumes hardly any additional energy. A magician has to select equipment and magical props and customize his or her preferred genre. The impact of magic tricks depends on the presentation as well as interpretation of the effect: “One is the ability to amaze and to make an effect, and to understand that the effect goes from instrument to technique and this is an important point because then it kicks you onto a trajectory that you have to develop. It's very important and then you get kind of naturalness to your performance. You can spontaneously be in a state where you know the performance.” (M6)

M9 reported feeling satisfaction when developing new tricks, especially when they are able to deceive colleagues with them. In addition, they believe that life as a magician is relatively free in nature without rigid daily routines: “I get a lot of satisfaction from inventing my own tricks. It is very satisfactory to me. I am pleased to lead this kind of … so called … free lifestyle with no schedules or routine based life. It all raises from this chosen profession and this hobby. These are the main things I enjoy. I also enjoy sessions with other magicians, the exchange of ideas. I get great satisfaction from being able to help someone solving a problem; it is a fantastic feeling when you notice that you've been able to help someone else for a change. That's where I get satisfaction, too.”

When magicians practice and acquire manual dexterity (hand skills), they try to imprint such sequences of gesture very deeply, often resulting in deep unconscious automatism. The interviews indicated that magicians practicing can be directly compared to that of musicians or acrobats as they spend countless hours trying to reach perfection in some techniques or body movement sequences (Jones, 2011). For a magician, refining the effect may be the most important, although an outsider may not be able to tell the difference: “From an outsider's point of view it may look like there's no difference but you yourself see the differences and then you develop it and look for it. Yeah, you can ask if it makes any sense. It's like… was it Leonardo Da Vinci who said that the divinity is in the details? Working on details, yeah they're developed throughout your whole life or until you get bored, that's a possibility as well. There's no such thing as perfection but you need to strive for it.” M15

There are different magic genres with their own distinctive subcultures, and practitioners try to establish hegemony of one form of magic performance over others. The interviewed magicians reported mastering a wide variety of magical genres. These included stage magic that involves manipulations (i.e., sleight of hand), stage illusions (based on huge props with animals or people), comedy magic (making people laugh), and mentalism (demonstrating seemingly superhuman mental powers). Most of the participants mastered various forms of close-up magic which is performed for a small group of people at close proximity. Such performances often use small instruments and objects and involve lots of audience participation. In magic competitions, such performances are assessed according to technical skills, showmanship, entertainment value, artistic impression, originality, and magic atmosphere.

#### Performance

Many of the interviewees highlighted the distinctive features of a magic performance; the audience expects to experience a miracle and the participants want to be surprised and astonished. When a magic trick is presented in the optimal way, the audience experiences a WOW effect. A magic show is a multifaceted performance where the magician must take into account several partial areas. The interviewed stated that both mental and manual skills are needed for successful magic performances. They emphasized the importance of manual dexterity to fluently perform tricks in various conditions and situations and to elicit maximum response in the audience. It was pointed out that the building of a performance depends on the magician's personality, style of performing, and the tricks which are performed, but it also depends on the audience. The magician has his/her own conscience about how he/she wants to create the illusion that the audience experiences. When a magician performs with lots of speech, he or she must be able to communicate with the audience and make the story understood. In shows built on the usage of birds or illusions, the chosen music and his or her coordinated body movements at the stage carry the show forward.

The interviewees highlighted the importance of the magician's personality and charisma. Many magicians are considered as having “magnetic” personalities that impress people around them, making their extraordinary and supernatural—magical—achievements appear plausible. They are also likely to have strong communicative competencies needed to persuade people to believe, at least partially, that something truly magical is occurring in front of their eyes. One of the respondents believes that personality and charisma are the most important factors in the work of a professional magician: “Everything else you can get through practise, but if you haven't got the personality, then it is just a waste of time. In addition, there is also: ambition, determination and courage to throw yourself into it.” (M15)

Some interviewees reported constructing a specific identity around their stage performance that shape and color their shows (Landman, 2013). These characters are often based on inspiring living models (a real person or a performative character). Initially, the character is often appropriated from some professional magician's performance. Later, the magician's own personality and deliberate building of the show start shaping and developing the character. In order to function well, the magician's personality and charisma, nature of the magic show, and the character performance should fit seamlessly together. The magicians deliberately build their own performance character and gradually develop it according to their evolving magic show and live interaction with various audiences, always working to improve it. “I can't be my normal self on the stage, I have to have a character. I need a stage personality, to whom the audience can identify themselves. There are so many things which I understood at the same moment. I started to create a character and it only took a couple of months when I got gigs and the whole system changed. I learned how to act while being on the stage. Then there were times when it didn't work when I was searching for my program, made it better using a lot of trouble in it, it was a great relief when everybody liked it so much.” (M15)

When working as a magician, the hope is to entertain, but also to earn a living. Simultaneously, however, stakes for a successful performance are very high because a brand must be shaped to create a reputation and generate new customers: “A gig well done: A hundred times more important than the money I get from it.” (M11) It was very important that event organizers are satisfied with the performance and expectations are exceeded for the arrangers as well as the audience. In this regard, the interviewees highlighted the importance of being able to cope with unforeseen and problematic performance situations. The audience and circumstances of performing may cause various surprises.

A magician has to utilize experience accumulated throughout a long professional career to be able to solve various challenging situations; however, the audience may not even notice that something special or out of the ordinary has taken place. Preparing and successfully completing a challenging performance provides its own endorphin kicks: “Of course the adrenaline, if you make the smallest change, everything feels quite different. You are always looking for some kind of kicks from it. Some go to the gym for getting endorphins, we go and seek it from our gigs.” (M16) Satisfaction is earned through gained insights and successful performing incredible improvisations: “I get professional satisfaction if some improvised trick has succeeded and I have invented a funny gag in it. It just flashed in my mind and I used it: it turned out to work fine. That's where professional satisfaction comes from.” (M4) This respondent also commented on the importance of improvisation in the capacity to negotiate problem situations: “It is essential to have the audience participate in. You may need to improvise in problem situations, for instance when something breaks down.”

#### Audience

The main focus for a magician is the performance in front of an audience. All the respondents highlighted the significance of the audience in the magician's work and in magician culture. One participant reported: “… [magic] doesn't exist without an audience. There is no magic without an audience, it is crucial. Even more important is to make your assistant enjoy being in front of the audience so that she/he doesn't feel uncomfortable.” (M12) The results of practice do not become concrete until the live performance. That is when the magician is able to see which effects and methods really work in practical situations. A magician will tailor their performance to fit the audience. For example, performing for children is very different from performing for a group of adults. A magician needs to identify the group's own language and ways of reacting and tailor his or her performance accordingly.

The audience expects to see and experience an exceptional performance. The magicians reported often being aware of the audience's expectations of them. A magician has his or her own expectations about the emerging performance and is scripting and planning the performance accordingly. There could, however, be unforeseen obstacles related to the audience and the performance stage; this highlights the importance of a professional magician's experience and improvisational capability. A magician has procedures, tools, and practices but needs to be able to modify the performance according to situational requirements. One participant reported, “You should have a good feeling about being on the stage. You are there and the audience is watching you. You don't necessarily do anything, but you know that the thing just runs nicely. You don't do just anything. The audience is looking at you and nobody gets bored. That is the greatest wonder you can ever do.” (M15)

There are many kinds of audiences and a magician has to be flexible and able to adapt his or her knowledge according to the situation. A magician needs to get the confidence of the audience, without trust he or she cannot get the expected response. Performances must be partially scripted and controlled in conjunction with situational improvisation to allow the magician to lead the audience in a desired direction. It is essential, in real-time, to be able to heed the audience's behavior and react to it continuously: “In sum, you should notice your audience and surroundings as perfectly as possible.” (M5) The audience's reactions and comments, surprising situations, and mistakes/errors of a performance challenge a magician. Improvisation is a productive way of functioning and mirrors professional competence.

The interviewees agreed that positive audience interaction and successful performances are the most important factors for experiencing professional satisfaction. A coherent performance emerges from intensive interaction between a magician and an audience: “Just the moment when [I am] standing before the audience … And it doesn't matter what I am doing there, the only thing that matters is how the people feel it, what they experience inside. It is what they take home with them, what they tell their children or grandchildren … or even what they write in their diaries or in their blogs or wherever… because that's all that matters. Of course I can see it in the professional way: when I walk to the stage it is my job… but it kind of cleans me of everything else, I feel totally free, when it goes at its best, free to everyone, free from all prerequisites, free from anything.” (M15)

A magic trick must be deliberately practiced until reaching a level where the technical performance hardly requires any physical or mental energy. The magician's performance differs from other performances in that the audience knows that the performer is trying to deceive them and deliberately lead them astray. A magician is not a true magician if his or her performance does not include any magical effects. The effect experienced by the spectator is the climax of any performance. The magician builds the trick by persuading the audience to see, hear, and think a certain way without understanding the method behind the trick. One respondent states: “I am a conductor and the audience is my orchestra.” (M15) The magic is born from a concept created by the magician that spectators try to interpret based on their own personal experiences. The spectators try to solve a riddle, but a cleverly built show does not allow them to rationally understand what they see: “Effect is the impact the performance has on the audience and includes not only the magical effect itself (e.g., disappearance, transformation, penetration, levitation, etc.), but also the emotional and post-performance impact on the audience.” (Landman, 2013)

During the performance, constant interaction between the performer and audience is imperative. All magicians emphasized the importance of the audience in their professional activity. One participant reported: “I pay attention to different individuals in the audience thinking about the next trick, and whom I am going to use as an assistant in it – and whom I am not going to use. I also try to imagine what kind of tricks different groups of people would be interested in. I try to watch all the time my audience to know their feelings. Improvisation is one important part of the show and that's why you've got to know the audience to see where it is heading to.” (M11)

One of the interviewees stated that observing the audience during a performance should be continuous to ensure optimal interaction between the performer and the audience: “I follow the reactions of the whole audience and try to conceive, in the earliest possible stage, if I need an assistant, whom I am going to choose. You always look at the audience and how they react in your performance. Usually, I try to go, in my performances, like on thin ice, and that's why I try to critically look at the audience to know where we are going in this thing and level.” (M10) To summarize, performing in front of audience is a crucial aspect of magic; competent magicians follow an audience reactions very carefully and tailor their activity accordingly.

### Reflecting and analyzing magical performance

The interviewees addressed their ways of self-reflection and of analysing their performances. The audience's reactions and feedback provided information about whether a new trick is a functional part of the overall performance and whether it needs to be refined or left out altogether. The magicians analyzed and reflected on their performances and the reactions of the audience during different stages of their work: “Performance is already rather demanding training; it is more reflecting on than training. I tried at least twice a week to film especially the novel illusion [of my own] and think what works and does not work in it, and could it somehow be improved.” (M4) Such an analytic process appears to be a central tool for the development of their expertise. One participant told the following: “I go to the backstage room and take off my jacket and sit down. I think and go through the performance: how did it go, did it work, or why didn't it work, what should I have done, what did I do wrong and what was working nicely. I kind of make a little analysis of how everything went. Yes I do my own analysis of the gig and pack my things and go to say thanks to the organizer of the performance and start my journey back home. And if it needs more replaying, I do it throughout the driving wondering why I am doing this kind of business. I stop for a cup of coffee and then drive home thinking about how I could have done my show even better. I also speculate about the length of the performance, was it too long or too short, were my choices of the tricks right or wrong and how could I make the performance better.” (M10)

Magician M7 does his first analysis immediately after the performance and speculates on the successes and failures: “I try to empty the gig and go through it already in the performance place. But the deeper analysis takes place in a silent and tranquil place. But the proper analysis takes place in the car… If the gig went well, you may not stay in the flow-experience… the next gig will start again from the zero point… If the gig went bad… You have to neutralize it again remembering that the next one will still start from the zero point.” Also M16 analyses his performance immediately after the show: “Yes, I go through the performance quickly, as soon as I come out from the stage or wherever I am. I think about it for a while like in a fast rewind mode speculating about how I succeeded, did the tricks go fine or did I make mistakes. Then, of course, I go to meet the organizer and put my things together saying thanks and goodbye. But after every performance, I do think and speculate about how everything went and what I said and try to find out how to improve my performance, or what I should change, and also how the audience has behaved. Every time I go through the performance myself or with someone else, if there is someone who has seen the show.”

Magicians who have a partner or an assistant go through the first debriefing and feedback immediately after the performance, either when dismantling equipment or during a return trip. They usually address those aspects that either went well or need improvement: “Earlier, it was very important to speculate and go through the program [when we were planning the program] to see what really is in it and to find out whether there were loose movements which we could drop out. We always had this personal meeting, I always trusted my assistant, and it was very important. Still, after all she follows the development of the situation between me and the audience, she is kind of a background person, as she is not the main hero on the stage.” (M2) The magician M1 also reflects after the show about the whole performance and things that happened: “After the gig everything depends on how it went and what kind of a gig it was, then we start to break it down. Me and my wife pack up the gear and throw a few comments about what was good and what went badly in our performance, what worked fine and what didn't, and where we should pay attention to next time.”

Four respondents (4 of 16) worked with animals, involving their own set of challenges. One of the participants commented: “Somehow you always go through the performance, especially when something goes a little wrong. Lately, it has happened with the birds. I just lost a few. They just simply got too old. The birds with which I started in the 80 s, they were so old that they just simply died. I lost many birds during a short period just though aging. It made a kind of a gap, because so many key-birds were missing at the same time.” (M15)

All magicians emphasized the importance of the audience in their performance because the magical effect emerges only in interaction with an audience. In order to perfect their performances, magicians need to constantly reflect on their magical programs, from individual trick to the overall performance, and gradually expand the repertoire of their activity.

### Creating novelty and making innovations

One theme of the interviews involved magicians' concerns with the pursuit of novelty and innovation. Magicians work in a rapidly transforming environment in which instruments, methods, and performance environment continuously change. We wanted to know why participants changed their tricks and performances and the process for creating new ones. The interviewees were asked to reflect on how they get new ideas, to what extent they transform their performances, and what aspects of their activity change.

New effects are integrated into the performance by incorporating a novel trick or program component to a prevailing show. The magician tests whether the routine needs changes or preparations and whether it is suitable for the overall program or should be abandoned. This helps to ensure that the entire show is under control, that the novel part fits in, and that the program develops gradually as a result of exploring and testing new elements. The show is perfected through refining its smallest details time and time again. M7 reported experiencing the greatest satisfaction when being able to create novelty and take things to new trajectories: “I guess it is inventing something new, bringing in some novelties, and when you notice that it works, it is not repeating the old thing again. There is nothing wrong in that, but you get the biggest kick when you take something totally new and see it working well; that's where the greatest satisfaction comes from, it is quite a different thing.”

A great deal of the participant talk related to their performances and consisted of programs of interrelated tricks. Magicians create performance products that are created and presented, so that tricks and magical performances may become commercial products. The respondents develop their expertise by reflecting on current programs, working through difficult aspects, and inventing new tricks and programs. Various external reasons elicit the creation of a novel act. An approaching significant performance and the development of new program force a magician to create something new. Also, a desire to meet the customer's novel expectations provides developmental pressures: “It was mostly that I was on fire because there was a new performance closing, or some TV show to make … I had to develop a lot of new material for them. Sometimes something might inspire me and I want to learn new things all the time, but I had so much pressure from the work to be able to fulfill all my deals and promises. So this is why I had to develop new tricks. It was obligatory.” (M1)

When magicians plan new performances, the old magic shows are assessed and reflected upon. Professional gratification is often obtained by having a very good feeling after a successful performance: “For selfish reasons, I reflected that people recognized your work, appreciated it, and recognized me as a successful magician.” (M6) Money is, of course, also an important motivation for developing performance and creation of novelty: “The money has been a good starter when something had to be done, but there is always the deadline and a date for everything. When you have promised to give a lecture in America on a certain date, you have to come up with new things to show and tell to the audience there. The working process starts from having a date, and creating something new before that date. The brain gets a message and starts working and something occurs, things start to develop, and inventions occur.” (M6)

Dissatisfaction with a routine can motivate the learning and practicing of new magic techniques; you need to change to avoid getting stuck in a rut of old practices: “Maybe it is a little dissatisfaction. I still have not found my own place or ways of expression in magicianship and work, or would I say as a transformer of magic.” (M12) Additionally, the will to explore, experience, find something new, and progress one's career can inspire change: “It is the need to experience new things, not to keep jamming in the same place and situation. You must try and find your own borders in magic … ” (M15)

#### Inspirations and pressures to create novelty

One motivation to create novel tricks and routines may start from encountering problems and challenges evolving into the need and desire to learn something new: “(New ideas) come from a strong will to develop when you really want to go forward in some field. It is like a burning fire. Then they just occur, of course you can get inspiration also from others, you can see a trick performed and think, that this point of view would be suitable for another trick … ” (M10) Ideas that are not immediately utilized will be reactivated later on enabling the creation of novel ideas: “Well, [new ideas] occur just by reasoning things up… ideas for performance entities, you just have to start solving the problems how to do it well … many technical solutions also occur when you start thinking about a new idea which again raises other new ideas and so on.” (M7)

Professional magicians report continuously seeking new ideas and inspirations for magic performance. They revealed that new ideas and fresh models of performance emerge in different ways and from various sources: “Just looking at other performers, which may be stand-up performers or other magicians or even comedy series in TV … Or even sitting in a cafe and looking at people passing by in different situations recognizing humorous potential of emerging situations occurs. It could be everyday life comedy or movies as well.” (M4)

Curiosity, interest, and engagement in the field motivate a magician and can be seen by an audience: “Most important is your own enthusiasm. You must love this business. In some stage, you get bored and you feel that you do not have the power to go further. Then you have at least one little new trick which you are excited about. It shows to the audience that you are on fire again.” (M4) M15 describes the mentally simulating tricks and performances in his mind: “They may just pop up in your head, or seeing an old trick and inviting a new way of performing it. It may start from music … sometimes I hear a piece of music and think that it would be great to do something using this music. Sometimes it starts from a situation: I think and start developing a trick suitable for a certain event.” (M15)

#### Between appropriation and stealing

Just like any other area of human activity, magic takes place at an interface between tradition and innovation. Magical activity relies on internalization of magical cultural tradition in conjunction with creative externalization involved in creating new tricks and programs. Knowledge creation often starts from observing and following other magicians' performances. M1 finds ideas by following other magicians and observing what they do: “In the way that I watched some Vegas shows like Cirque de Soleil and other magicians, I stole and copied their performances just like all the others did.” (M1) Social learning by imitating and modeling colleagues' performances is commonly used as a way of developing new performances.

It is difficult to tell where different tricks stem from in magicianship because the origins are almost impossible to be found: “Of course stealing ideas from others is common (laughing) and changing them so that audience would not notice what had happened. Sometimes, but quite seldom, a pure idea may raise when you are planning something and you find out a new way of executing the idea. An accident, or a surprising event happens, it is like Picasso said, I don't seek, I find.” (M6) Experienced magicians will observe their colleagues' performances and reflect on the audience reactions to develop new performance ideas.

The interviewees pointed out that innovation occurring in magic activity often involved restructuring and recombining elements and aspects of already existing tricks and performances: “I can join other's tricks together and create unforeseen entities. This is the way to create something out of almost nothing.” (M3) In many cases, a magic effect is borrowed and worked out from an original way of implementing it. Developing new magic effects is very challenging: “Inventing a new effect is the most difficult. Almost every time you have an effect, you can find out the method to carry it out, as well.” (M9)

By utilizing and applying old methods concurrently with contemporary methods and instruments, a new creation may materialize: “The best way of creating new things is through connecting old things (tricks) which no one has used for decades. This is the way I find new ideas, through something which already exists.” (M11) Respondent M16 reported that he did not find inspiration from following other magicians' performances directly: “I don't get any ideas from conferences of magicians. Pretty seldom I find anything from other magicians' performances either. I get new ideas more indirectly from various cultural sources and happenings: I get quite many ideas from movies, journeys and museums, discussions with really experienced performers. I listen to their stories – all ears: Billy McComb, Reijo Salminen was one of the most important. Books. Leonardo Da Vinci: Complete Works of Leonardo Da Vinci. When you are on a holiday trip where your body rests, the mind often starts to gallop. It happens in a strange culture with no mobile phone around ringing all the time.”

The interviewees agreed that it is inappropriate to copy tricks or program components directly from fellow magicians. When taking inspiration from another magician's trick, it must be modified and developed to transform and adapt it. Borrowing other magicians' tricks or programs are unacceptable and seen as “stealing.” There was extensive discussion about stealing other magicians' tricks, stories, and program components on a Finnish magician's website (TaikaWeb) that resulted in practically all Finnish magicians signing a commitment to respect other magicians' copyright, original innovations, and creative achievements. Unlike the music or movie industry, the law does not protect magicians and such a collective commitment appeared to be needed. Simultaneously, it was acknowledged that everyone receives inspiration from the magic culture and each other's performances, however borrowed ideas and elements must be creatively adapted and extended.

Faster transmission and sharing of knowledge through the Internet has affected the concurrent requirements for magical activity. It is easier to get access to magic knowhow, have wider audiences, and build national and international reputations much more effectively than before. Also, the magic world and culture have changed from last century's secretive and mystic magic, to become more public, open, and multi-faceted in nature: “Well, it is so that when you read these old books, you have to be able to see them in the context of the time. You must think that ‘OK it was done in the 50 s and the world was different in those days.’ They had time to take, for example, seven things in a blindfold trick and go through them all one by one. Now if you would do it for example two times, the audience would be bored, Can't he do anything else?” (M5)

Sometimes performances are developed through brainstorming by groups of magicians, which may generate creative ideas to improve quality and create new tricks. Social sharing takes place when receiving inspiration from other magician's shows and transforming their tricks to one's own performance. M7 reported experiencing satisfaction when he/she was able to create a novel trick and take things to new trajectories: “I guess, it is inventing something new, bringing in some novelties, and when you notice that it works, it is not repeating the old thing again. There is nothing wrong in that, but the biggest kick you get when you take something totally into new tracks and see it working well; that's where the greatest satisfaction comes from, it is quite a different thing.”

To conclude, successful magicians invest a great deal of time and effort to create original and innovative magical programs. Although they get inspiration from their fellow magicians and capitalize on cultural achievements in the field, they are oriented to creatively adapt and extend such inspirational sources. In order to keep their levels of expertise, and often raise it, successful magicians must deliberately work at the edge of their competencies and break boundaries.

## Discussion

The present study addressed various aspects of professional activity of professional Finnish magicians. The interviewees (*N* = 16) were selected because they were nominated by their peers as the most highly regarded magicians in Finland. Qualitative analyses of the interviews revealed that magic is a unique professional field; in spite of requiring years of deliberate practice, practitioners of the field have hardly any formal training. The time from initial contact with the magical culture and becoming a professional expert in the field varied from 7 to 23 years. As there is no formal training system, most of the development takes place through informal communities of practices (Lave and Wenger, 1991). For that reason, creating, keeping up, and developing personal social networks with other magicians and professional experts from various fields play an important role. Cultivation of their expertise takes place with tremendous personal effort facilitated by participation in informal networks. Magicians are entrepreneurs who have to make their living by personally creating their own brand and reputation in a very small and competitive market. In order to survive professionally, the magicians have to master various domains of magic and cultivate versatile performance skills.

Magicians can be very peculiar, yet are often compared with other professionals like actors, musicians, or stand-up comedians. Some of the same characteristics can be found in these professions, but there is no other profession where it is essential to preserve trade secrets. Pursuit of magical performance consists of ingeniously integrated magic tricks that together create an impressive and sometimes astonishing show. Once the tricks are learned, they provide a flexible basis for creating situationally adequate and contextually varying performances that are adapted to specific features of the audience in question. Each trick may be seen as a routine activity sequence that can be triggered with appropriate situational cues, hints, and deliberation.

Magicians calculatingly utilize various techniques for misleading the audience, such as forcing, misdirection, and illusion; the audience observes the magical effect, but the method for the trick is kept secret. Our data revealed that magicians do not willingly reveal the tricks of their trade with anyone beyond a trusted apprentice or colleague^1^. Consequently, it is understandable that the interviewees did not talk much about their tricks or associated technical performance, but concentrated on more general reflections of their performances and shows. They shared experiences of preparing, conducting, and reflecting on their magical performance. They developed expertise by reflecting on current programs, working through difficult or not so optimal aspects of it, and developing new tricks and programs. Today, the revolution of audio/visual and digital technology provides new tools to develop tricks, new channels for performance, and new ways of documenting the performances.

For many interviewees, the audience was the most important aspect of their activity. They were willing to do almost anything to entertain the audience. Toward that end, every interviewee reported investing a great deal of effort reflecting on their performance. A successful performance involves moment-to-moment improvisation combined with well-scripted elements. The interviewees reported frequently adapting their performance according to opportune moments and situations emerging across real-time interaction with their audiences. In many cases such enacted adjustments affected the direction of their subsequent performance. Over time, magicians need their repertoires of tricks to be able to adapt to varying contexts. It may be necessary to move to a neighboring area of magic and learn to hybridize very different kinds of tricks as components of a new performance program. One of the interviewees pointed out that pursuing an original line of professional magic may require seeking inspiration from beyond the magic scene, such as theater, opera, music, visual arts, and observing people. Pursuit of innovations requires a strong motivation.

In many cases, external pressures of performance, crises, failures, challenges, seeking personal advantages, or competition may elicit creation of novelty. When earlier performances have become routine, degrees of freedom from the magician provide ample opportunities for knowledge creation. In order to maintain expertise in the rapidly changing world, magicians cannot rely on an old repertoire of tricks but need to function as adaptive experts (Hatano and Inagaki, 1992; Bereiter and Scardamalia, 1993) who invest a part of their resources in learning and creating new tricks. Integrating different tricks and practices often provides unforeseen creative opportunities, fostering innovation and transformation of performances, which expand the magician's repertoire. Combining unexpected routines may also inspire curiosity for developing new ideas. This creation of new effects may come from a desire to investigate or explore novelty-seeking opportunities, or merely a happy coincidence.

Many of the interviewees talked about borrowing and stealing from other magicians. In many cases, a magic effect is copied and developed in one's own way of implementing it. The interviewees were concerned about using tricks, program components, or whole programs from other magicians without acknowledgement. Most magic tricks are not protected by copyright law. This has been a longstanding problem in magic. Most magicians are reluctant to patent their tricks because doing so would give the secret away. During a magic show, magicians very rarely acknowledge the writer or creator of a trick, which is in great contrast to other domains (e.g., music, film, or literature). The interviewees discussed the efforts of the Finnish magic circle to establish ethical norms for professional conduct in magic. Acceptable social sharing involves getting inspiration from other magicians and transforming their tricks by adapting them to one's own performance.

The results revealed that a professional magician's expertise is particularly apparent in challenging and problematic situations. A skilled magician uses the talents and competencies gained through years of experience to solve a problematic situation creatively without drawing attention to the special circumstances. Their professional competence relies on a rich repertoire of tricks, program components, and orienting stories which can be adapted to diverse situations. Their professional expertise likely builds on both procedural skills and declarative knowledge, integrating practical and conceptual mastery of their trade. The present data did not, however, reveal other evidence of conceptual knowledge other than the participants' fluent ways of talking about various aspects of their craft and associated performances.

This study focused on examining the professional expertise of highly regarded Finnish magicians. The nationally representative group of magicians is considered an appropriate sample of the magic community in general. A limitation of the present exploratory investigation was that only the participants' verbal reports and retrospective reflections regarding their professional practices were addressed. Although this is justifiable when pursuing one of the few studies of professional magic activity in Finland, it should be taken into consideration while interpreting the results. The participants are likely to provide reliable and valid accounts regarding only those aspects of their activity that rely on deliberate and conscious information processing, such as preparing, managing, and reflecting on their performances. Tacit and automated aspects of motor performance in magic tricks were not addressed in the interviews. The data do not directly represent magic practice, but rather the participants' meta–level reflections.

All participants had long careers and were interviewed only once. Information about various stages in the development of their expertise provided only a partial and fragmentary picture of the actual process (Reis and Gable, 2000). It would be desirable to carry out future investigations by repeatedly documenting various aspects of a magician's learning, activity, and development. It is possible that participants' interpretations of socially desirable aspects of professional magical activity have colored their interview responses. The interviewer was himself a magician; the participants could have revealed different aspects of their professional activity to another kind of investigator. Nevertheless, the respondents were professionally highly-regarded magicians and their interviews provided very coherent and comprehensive views about various aspects of their activity.

Research on magical expertise is provoking increasing international attention, scientific discussion, academic research, and artistic activity. The results of the present investigation assist in understanding and explaining the nature of magical expertise, the systematic development of magicians' training, the adoption of creative practices that support the continuous development of expertise, the sharing of magical knowledge and competence, and the utilization of social and cultural capital for professional magicians and mentors. From a wider perspective, this study may contribute to the broad field of expertise and skilled performance. It appears that understanding expertise in such a specialized area as magic, once better understood, may have implications. The term “expertise” has been dominated by such arenas as medicine, and a wider set of data, from an area with its particular requirements, may provide for strengthened foundations for expert research.

### Conflict of interest statement

The authors declare that the research was conducted in the absence of any commercial or financial relationships that could be construed as a potential conflict of interest.

## Footnotes

^1^Most magicians disapprove of exposure for the sake of exposure (e.g., Swiss, 2001), but are happy to discuss their methods with non-magicians, if there is a scientific (discussion with scientist) or artistic purpose (film/theater producers).
