# Supplementary material for: Expertise among professional magicians: an interview study
Source: Front Psychol. 2014 Dec 23;5:1484. doi: 10.3389/fpsyg.2014.01484 (PMC4274899; doi:10.3389/fpsyg.2014.01484)
Supplement: Supplementary file 1 [file DataSheet1.DOCX]

### Appendix

**Table 1** The trajectory stages of professional magicians and summary of the interview data analysed.
